# Early adoption of image-guided histotripsy therapy in interventional oncology: challenges and opportunities in the United Kingdom

**DOI:** 10.1093/bjr/tqag047

**Published:** 2026-03-12

**Authors:** Helen Hoi Lam Ng, Vinson Wai-Shun Chan, Lewis Howell, Taha Shiwani, Jim Zhong, Jacqueline Brandon, Adel Samson, James Chandler, James McLaughlan, Tze Min Wah

**Affiliations:** Department of Diagnostic and Interventional Radiology, St. James’s University Hospital, Leeds Teaching Hospitals NHS Trust, Leeds LS9 7TF, United Kingdom; Leeds Institute of Medical Research, Faculty of Medicine and Health, University of Leeds, Leeds LS2 9JT, United Kingdom; Department of Diagnostic and Interventional Radiology, St. James’s University Hospital, Leeds Teaching Hospitals NHS Trust, Leeds LS9 7TF, United Kingdom; Leeds Institute of Medical Research, Faculty of Medicine and Health, University of Leeds, Leeds LS2 9JT, United Kingdom; School of Computer Science, Faculty of Engineering and Physical Sciences, University of Leeds, Leeds LS2 9JT, United Kingdom; School of Electronic and Electrical Engineering, Faculty of Engineering and Physical Sciences, University of Leeds, Leeds LS2 9JT, United Kingdom; Department of Diagnostic and Interventional Radiology, St. James’s University Hospital, Leeds Teaching Hospitals NHS Trust, Leeds LS9 7TF, United Kingdom; Department of Diagnostic and Interventional Radiology, St. James’s University Hospital, Leeds Teaching Hospitals NHS Trust, Leeds LS9 7TF, United Kingdom; Leeds Institute of Medical Research, Faculty of Medicine and Health, University of Leeds, Leeds LS2 9JT, United Kingdom; Department of Diagnostic and Interventional Radiology, St. James’s University Hospital, Leeds Teaching Hospitals NHS Trust, Leeds LS9 7TF, United Kingdom; Leeds Institute of Medical Research, Faculty of Medicine and Health, University of Leeds, Leeds LS2 9JT, United Kingdom; School of Electronic and Electrical Engineering, Faculty of Engineering and Physical Sciences, University of Leeds, Leeds LS2 9JT, United Kingdom; Leeds Institute of Medical Research, Faculty of Medicine and Health, University of Leeds, Leeds LS2 9JT, United Kingdom; School of Electronic and Electrical Engineering, Faculty of Engineering and Physical Sciences, University of Leeds, Leeds LS2 9JT, United Kingdom; Department of Diagnostic and Interventional Radiology, St. James’s University Hospital, Leeds Teaching Hospitals NHS Trust, Leeds LS9 7TF, United Kingdom; Leeds Institute of Medical Research, Faculty of Medicine and Health, University of Leeds, Leeds LS2 9JT, United Kingdom

**Keywords:** Histotripsy, Interventional Oncology, Interventional Radiology, Medical Technology, United Kingdom, Ultrasonic Therapy

## Abstract

Histotripsy represents a paradigm shift in interventional oncology (IO) as the first non-invasive, non-ionizing and non-thermal ultrasound-based ablation technology available for cancer therapy. Compared with thermal ablation techniques, the advantages of histotripsy include tissue-selective ablation near critical structures, reduced collateral injury risk, and treatment which is unaffected by the heat sink phenomenon, ensuring predictable treatment margins. Ultrasound technology can be constrained by tissue attenuation depending on the depth of the target; however, the early phase feasibility and pivotal trial results have been promising for its application in liver cancers, with emerging translational trials in renal and pancreatic cancer. In the United Kingdom, 2 well-established IO sites have participated in the pivotal #HOPE4LIVER Trial that led to approval by the US Food and Drug Administration in liver tumours therapy in 2023 and obtained Medicines and Healthcare products Regulatory Agency Unmet Clinical Need Authorisation for treatment of liver tumours in United Kingdom (April 2025) via the Innovative Devises Access Pathway. The global-first feasibility in renal cancer (CAIN trial) was also led by the United Kingdom and completed in April 2024. This review provides an overview of histotripsy and highlights the clinical challenges in early National Health Service (NHS) adoption such as the learning curve for operators and teams, regulatory processes, and synthesis of health economic evidence required for wider NHS commissioning. The review will also discuss the future directions of histotripsy, including combination immunomodulatory therapies, highlighting the need for continual national collaboration for successful integration in the NHS. Successfully integrating this technology into the NHS hinges on a unified national effort to navigate the clinical, regulatory and economic hurdles, ensuring its benefits reach patients nationwide.

## Introduction

Medical interventions are undergoing a notable shift towards less invasive procedures, with numerous diseases amenable to minimally invasive approaches, facilitated by improved image guidance. Percutaneous needle-based thermal and non-thermal ablation techniques, including radiofrequency (RFA), microwave (MWA), cryoablation and irreversible electroporation (IRE), are widely described and have demonstrated success across diverse applications.^1^

Image-guided histotripsy ultrasound-based therapy represents a paradigm shift in modern medicine. In interventional oncology (IO), this is the first non-invasive (needle-less), non-ionizing and non-thermal ablation technology, using primarily mechanical forces, rather than thermal effects^2^ to treat cancer. This technique delivers high-amplitude, short-duration ultrasound pulses to induce acoustic cavitation in the focal region, creating a highly localized “bubble cloud” where tiny nanometre-scale gas pockets expand up to 100 000 times their original size, compressing adjacent cells before violently collapsing within a few hundred microseconds.^2^ This imparts significant stress and strain on the targeted tissue, and over a number of cycles, the forces imparted by the expansion and collapse of these microbubbles destroy the tissue at a subcellular level, resulting in liquefaction to form an acellular lysate.^3^ In the literature, there are 2 dominant mechanisms which have been proposed for histotripsy: acoustic cavitation and boiling. Acoustic cavitation histotripsy uses high acoustic pressures to cause mechanical tissue destruction based on inertial cavitation, while boiling histotripsy uses thermal effects to achieve rapid boiling, followed by secondary cavitation effects, based on shock scattering of subsequent ultrasound pulses.^4^^,^^5^ For the purpose of this review, we refer to acoustic cavitation histotripsy, as this is currently the only histotripsy technique applied in humans.

Histotripsy’s unique combination of physical, biological, and immunomodulatory effects makes it an exciting technology to explore in humans. This is particularly promising considering its non-thermal, precise, and non-invasive nature. This means it could theoretically reduce the risk of complications for patients who are unsuitable for surgery or conventional ablation due to tumour location close to vital structures, comorbidities, or frailty. This approach has been examined in various pre-clinical contexts^3^^,^^6^ prior to clinical translation in human trials.

The United Kingdom has a rich history of pioneering impactful medical breakthroughs, from the smallpox vaccine^7^ to the discovery of DNA’s double helix.^8^ Today, the UK boasts a fully integrated health system, the National Health Service (NHS), and is both a catalyst and a gatekeeper for medical innovations. The NHS Long Term Plan emphasizes the importance of accelerating the adoption of proven, cost-effective innovations to improve patient outcomes while ensuring sustainable healthcare delivery.^9^ Histotripsy aligns with these goals by offering a non-invasive alternative to conventional ablations and traditional surgical interventions, potentially reducing hospitalization time, improving patient experiences and clinical outcomes.^10^ Histotripsy is at a pivotal stage in the United Kingdom, as with the first cases performed as part of clinical trials in liver and kidney tumours, namely the #HOPE4LIVER^11^ and CAIN trials.^12^ The #HOPE4LIVER trial has shown the treatment is safe and effective in treating primary and secondary liver tumours.^11^^,^^13^ In October 2025, Addenbrookes, Cambridge University Hospital, became the first NHS site to acquire histotripsy technology through a Li Ka Shing (LKS) philanthropic donation and treated the first NHS patient.^14^^,^^15^ The initial experience of the CAIN trial has shown promising early safety and efficacy outcomes in treating renal tumours at 1 year follow-up,^16^ with full CAIN trial results awaiting publication. These opportunities for UK healthcare institutions to deliver clinical trials to evaluate this innovative technology for translation into the clinical setting have provided invaluable learning for participating institutions. This has concurrently allowed the UK population to access medical innovations, which will set the stage for further exploration of clinical applications and facilitate the adoption of histotripsy into clinical practice.

This review aims to highlight the technological advantages of image-guided histotripsy and examines the current clinical applications, including the early UK landscape within IO. The challenges and opportunities of incorporating histotripsy into the NHS are also explored, drawing on the current NHS priorities and ongoing research activities. The goal is to inform interventional radiologists and other clinicians about considerations for adopting histotripsy into NHS practice.

## Advantages of histotripsy for clinical application in cancer therapy

Histotripsy was developed primarily for solid tumour ablation in soft tissues, due to its unique features and advantages over traditional ablation and surgical techniques.^3^^,^^17^ Unlike thermal high-intensity focused ultrasound (HIFU), which uses continuous wave ultrasound to heat and destroy targeted tissue via coagulation necrosis,^18^^,^^19^ histotripsy is a primarily non-thermal technique, employing acoustic pressures an order of magnitude higher than thermal HIFU, delivered in short pulses. This provides the pressure needed to induce cavitation in the focal region, generates cavitation fields, or “bubble clouds,” that disrupt cellular structures in the targeted tissue, with minimal off-target effects.^3^ Histotripsy is a threshold effect, dependent on negative peak acoustic pressure, which creates sharp boundaries (<100 µm) between lysed and intact tissue.^20^^,^^21^ This non-thermal mechanism is advantageous, as the main limitation of thermal HIFU is that it is challenging to precisely control thermal effects due to tissue inhomogeneity, heat sink effects, and changes in tissue properties during treatment, which can lead to over- or under-treatment.^22^^,^^23^ Additionally, this mechanical mechanism allows one of histotripsy’s most remarkable features: tissue-selective ablation capability.

Different tissue types exhibit varying susceptibility to damage from histotripsy, resulting in a differential threshold for mechanical destruction.^3^ Treatment of lesions in close proximity to critical connective structures, such as bile ducts, large blood vessels, and the renal collecting system, is made possible due to their high ultimate tensile strength compared with solid tumour or organ tissue,^24^ providing selective resistance to mechanical damage from histotripsy. This reduces risks of collateral injury, as demonstrated in animal models.^25^ These tumours, particularly those in the caudate lobe, which is close to the inferior vena cava (IVC), are traditionally challenging to treat. This is because conventional ablation requires difficult percutaneous needle access, and the tumours are often in locations that are not amenable to surgery.^10^^,^^26^^,^^27^ Furthermore, conventional ablation or surgical resection of these complex tumours can often cause collateral damage to vital structures such as the ureter, collecting system or the IVC.^28–31^

Pre-clinical studies have shown that histotripsy can activate the adaptive immune response, contributing to tumour suppression and potential abscopal effects.^32–34^ It is thought that the acellular homogenous liquid from histotripsy, which can be reabsorbed by the body, contains intact proteins and tumour antigens that may act as immune stimuli.^35^ Further research is required to assess the presence of these effects in humans as well as the potential clinical benefits of combination therapies in generating a robust immune response against tumours.^34^

Traditional thermal ablation methods (RFA, MWA, cryoablation) are susceptible to the heat sink phenomenon, where heat is dissipated due to blood flow, resulting in lower treatment efficacy for tumours located near vessels over 3 mm in diameter.^36^ Irreversible electroporation is a non-thermal technique which applies short pulses of electricity to increase cell permeability, leading to apoptosis and cell death.^37^ IRE has been utilized clinically for different tumours with multiple studies demonstrating an acceptable safety profile and high efficacy rates.^38^^,^^39^ Despite its non-thermal profile, there has been a report of possible thermal injury,^29^ highlighting the need for careful consideration of electrode configuration. Histotripsy’s non-thermal mechanism is not subject to the heat sink phenomenon, resulting in a predictable and consistent treatment margin near vessels, as demonstrated in porcine models.^20^ While further direct comparison evidence is needed, histotripsy may have a potential advantage over traditional thermal ablation methods in minimizing the heat sink effect.

Radiation can cause biological harm to the human body, including cancer and skin damage.^40^ For example, a typical percutaneous kidney cryoablation exposes the patient to an average effective dose of 40 mSv, which is equivalent to a 0.2% (1 in 500) lifetime fatal cancer risk.^41^^,^^42^ Similarly, a typical liver stereotactic ablative radiotherapy exposes the patient to about 200 mSv effective dose of radiation, which is equivalent to a 1% (1 in 100) lifetime fatal cancer risk.^42^^,^^43^ Furthermore, stereotactic ablative body radiotherapy for kidney cancer is associated with up to 84% of adverse event rates, including vomiting, colonic obstruction, nausea, diarrhoea, dermatitis, haematuria, colitis, and others.^44^ These are likely deterministic side effects of radiation.^40^ Histotripsy has the benefit of avoiding these risks as a procedure that uses non-ionizing radiation, namely ultrasound.

Compared to surgical resection, traditional ablative therapies have a lower risk of overall complications including pulmonary, wound-related and cardiovascular-related complications, among others.^45^ Given the non-invasive nature of histotripsy, which does not require any percutaneous probe insertion into the treatment site, there is less risk of localised bleeding or infection related to probe insertion.^46–48^ The initial #HOPE4LIVER trial had a total of 44 patients with 49 liver tumours treated, with a 95% technical success rate and a 7% major complication rate.^49^ Primary technique efficacy was achieved in 83% of patients at 30-day follow-up. Latest follow-up at 12 months has shown 90% local tumour control, similar to the current standard of care for locoregional therapies.^13^ Post-regulatory approval and during the early market adoption phase, a recent analysis of the United States clinical experience of 230 liver histotripsy cases revealed a complication rate of only 5.2%, and a major complication rate of 1.3%,^10^ which is favourable compared to the 30-days complication rate for other locoregional therapies (>12%), described from pooled analyses.^50^^,^^51^ Therefore, patients who are unable to undergo surgery or image-guided ablation will be able to consider this as a safe alternative.

Despite its advantages, histotripsy presents inherent limitations. The use of ultrasound provides real-time histotripsy visualization, but some tumours might be obscured by adipose tissues, overlying bowel gas, ribs or lungs.^52–54^ Ultrasound attenuation will also mean that the efficacy of histotripsy is constrained at deeper tissue depths. These factors make it challenging to target deeply located lesions, a difficulty often encountered in patients with larger body habitus. There is ongoing research in aberration correction to overcome these challenges.^55^ Comparison of histotripsy and other conventional ablative therapies and surgery are outlined in Table 1.

**Table 1:** Comparison of different major ablative methods and surgery.

Feature	Histotripsy	**High-intensity focused ultrasound (HIFU)**	**Traditional image-guided ablative modalities (RFA, MWA, CRYO)**	Stereotactic body radiation therapy (SBRT)	Surgery
**Mechanism**	Mechanical cavitation (non-thermal)	Thermal and/or mechanical	Thermal. RFA and MWA using heat and cryoablation using extreme cold	High-dose radiation to damage tumour cell DNA and tumour vasculature	Physical resection
**Precision**	High precision with sharply demarcated lesions	High precision but susceptible to heat sink and may affect nearby structures	Precision limited by thermal diffusion (“heat sink”). Margins can be less predictable	High precision	Direct visualization enabling clear margins upon removal. Can be challenging for lesions near critical structures or multiple lesions
**Heat sink effect**	Unaffected	Moderate to low	High to low dependent on modality (or none in IRE) and tumour proximity to vessel	Unaffected	Not applicable
**Invasiveness**	Non-invasive	Non-invasive	Minimally invasive (single or multiple needle insertions)	Non-invasive	Invasiveness dependent on approach
**Radiation risk**	None	None	Minimal when performed under CT guidance; None under MR guidance	High. Short- and long-term side effects	None other than pre-operative imaging (CT) for treatment planning
**Risk of complications**	Discomfort, damage to nearby tissues (rare)	Post-operative pain. Possible skin burns	Bleeding, infection, damage to adjacent organs (eg, bowel, diaphragm), pain, nerve injury, and tumour seeding along the probe tract (rare)	High rates of adverse events, including vomiting, abdominal pain, bowel obstruction, nausea, diarrhoea, Fatigue, gastritis, dermatitis and more.^56^	Higher risk of overall complications including pulmonary, wound-related, cardiovascular-related, intestinal complications, bleeding.
**Recovery time**	Minutes to hours	Hours to days	Hours to days	Hours to days	Days
**Limitations**	Currently requires direct ultrasound visualization; limited in cases near gas-containing organs	Difficulty treating deep seated tumours; accurate monitoring of real-time temperature, imprecise targeting due to temperature gradients, limited in cases near gas-containing organs	Difficulty treating tumours near critical structures with heat-based modalities; access issue if path to tumour is blocked by critical structure such as bowel/vital structure. May not be as effective as other modalities in treating tumours above a certain size	Difficulty treating tumours near radiosensitive structures. Complex treatment planning and multiple visits for patients	Relatively invasive

## Current clinical translation of image-guided histotripsy

Histotripsy was first explored for human use in the prostate. Canine studies showed that the liquid consistency after prostate histotripsy facilitated urethral drainage and effectively debulked the prostate,^56^ showing potential for treating benign prostatic hyperplasia. However, these results were not successfully replicated in humans,^57^ postulated to be due to the non-uniform stiffness exhibited by the nature of tough prostatic tissue and fibrosis.^58^ Despite this, a significantly reduced International Prostate Symptom Score of 44% at 6 months was observed, highlighting the potential for clinical benefit.^57^ Its use has also been explored in prostate cancer,^59^^,^^60^ but has not yet been translated to human use with concerns for damage to the urethra and blood vessels.^3^

**Figure 1:**
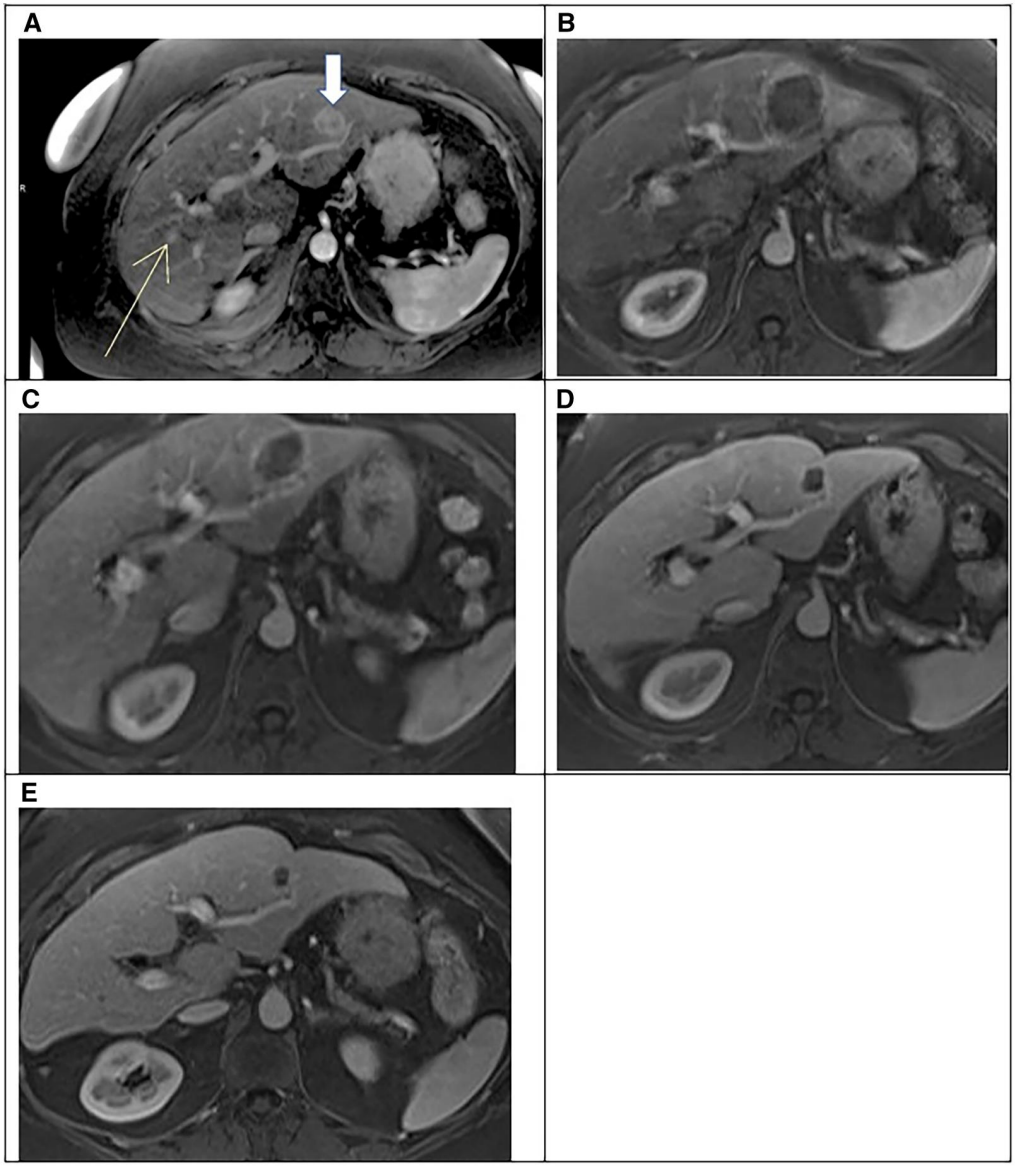
Images from patient of #HOPE4LIVER study,^11^^,^^13^^,^^49^ showing (A) a 2-cm hepatocellular carcinoma in segment II (wide arrow), which was treated with histotripsy and a 1.5-cm hepatocellular carcinoma in segment VIII (thin narrow), which was treated with image-guided microwave ablation. Follow-up MRI at (B) 1-day post-histotripsy showing a non-enhancing treatment zone. Subsequent follow-up at (C) 1 month, (D) 6 months, and (E) 1 year showing progressive involution of the treatment zone.

**Figure 2:**
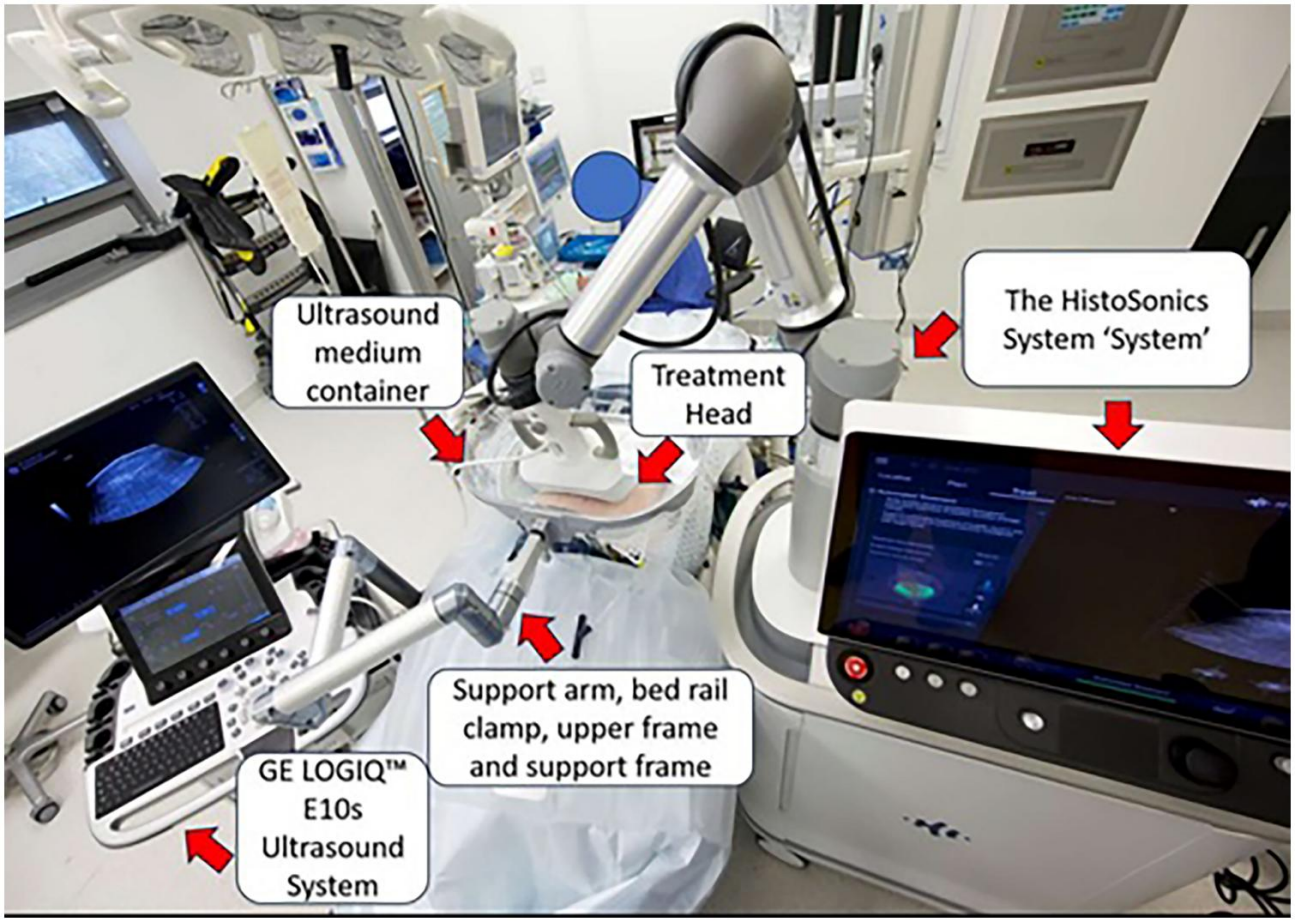
Image taken from Wah et al.^12^ Overhead view of the HistoSonics system components during histotripsy for treatment of kidney cancer from the CAIN trial. The GE LOGIQ™ E10s Ultrasound System and probe are integrated for tumour targeting, planning, and monitoring treatment. The treatment head contains both the histotripsy therapy transducer and an integrated ultrasound imaging probe. A multi-joint articulating support arm attaches to the bed rail, holding a reservoir that contains the ultrasound medium (degassed water) in position above the patient. A single-use patient membrane is inserted into the reservoir frame, creating a container for the ultrasound medium that conforms to the patient’s body, ensuring acoustic coupling between the treatment head and the patient.

The leading clinical application worldwide for image-guided histotripsy is currently in primary and metastatic liver tumours. The first in-human trial, THERESA^61^ (NCT03741088), evaluated the safety and technical effectiveness of hepatic histotripsy. The UK’s histotripsy journey began through participation in the subsequent clinical trial in September 2021, the #HOPE4LIVER Trial (NCT04573881), led by Leeds Teaching Hospitals NHS Trust (LTHT) working in collaboration with Newcastle upon Tyne Hospitals NHS Foundation Trust (NuTH). This collaborative multi-centre pivotal liver histotripsy trial in the United States, Europe, and the United Kingdom, evaluated the safety and efficacy of histotripsy for primary or metastatic liver tumours in cases where other local-regional therapies were unsuccessful or not tolerated. The results of this trial from a total of 44 patients with 49 liver tumours were described in the previous section.^49^ One of the first treatments in the United Kingdom, at LTHT, took under 7 minutes to complete and had excellent mid-term oncological durability, as shown in Figure 1. The patient reported minimal pain and minimal disruption to daily activities from 1 day post-histotripsy.^62^ The positive results of the #HOPE4LIVER pivotal study^49^ enabled the US Food and Drug Administration (FDA) to approve histotripsy for liver tumours in October 2023,^63^ with subsequent rollout of the HistoSonics Edison™ System at multiple centres across the United States and in select international locations.^64^ The industry-sponsored post-market, observational, single-arm, non-randomized, prospective BOOMBOX Master study will provide further insights into the effectiveness and safety of the HistoSonics Edison™ histotripsy system for the treatment of different types of liver tumour.^65^  Figure 2 shows the layout and components of the Edison system from HistoSonics during a histotripsy procedure,^12^ which recently featured in the 10 Year Health Plan for England.^66^

Beyond the liver, early studies have explored other cancer targets, notably renal cancers. The first-in-human kidney histotripsy case was performed in March 2023 at LTHT as part of the CAIN trial^12^^,^^16^ (NCT05432232). The CAIN trial is a prospective, multi-centre, single-arm feasibility trial,^12^ which aims to evaluate the technical success and safety profile of the HistoSonics system for the treatment of solid renal tumours. This first case at LTHT highlights the necessity of a multi-disciplinary team approach with careful patient selection. Pre-procedural ultrasound assessment and planning are crucial to determine the most appropriate treatment approach, tumour depth, and optimal patient positioning. The histotripsy treatment head is then placed directly above the target to provide the shortest therapy head to target distance. Respiratory motion management is also vital to ensure the tumour being targeted is completely included within the field of therapy throughout the histotripsy treatment cycle. At 1-year follow-up, this first patient had no evidence of residual or recurrent disease within the zone of ablation and only an area of involution.^16^ Recruitment is now completed, and the results are awaited.

In February 2024, HistoSonics Edison™ Histotripsy System was 1 of the 8 innovative technologies being awarded the UK Innovative Device Access Pathway (IDAP) Pilot Program for fast-track regulatory approval.^67^ In April 2025, the Medicines and Healthcare products Regulatory Agency (MHRA) granted Unmet Clinical Need Authorisation for liver tumours.^68^ This has allowed the Edison™ system to have early limited market access, meaning that NHS Trusts are allowed to deploy the Edison™ system under a tightly regulated framework. In October 2025, Addenbrookes, Cambridge University Hospital, became the first NHS site to acquire histotripsy technology through LKS philanthropic donation, and treated the first NHS patient.^14^^,^^15^

The UK-led CAIN trial^12^ has emerged directly from strong partnerships between the NHS, industry, and academia. This enabled a global-first medical innovation translation in renal cancer through clinical expertise in the NHS and sponsorship by the industry partner. The early experience from the CAIN trial has provided the baseline for commissioning of the pivotal #HOPE4KIDNEY trial (NCT05820087) in the United States, which aims to assess safety and efficacy in primary renal tumours for FDA approval. It can be expected that further collaborations between NHS Trusts and industry can expedite wider early adoption of innovative histotripsy in the UK and allow faster research and clinical translation into both cancer and non-cancer therapy in various diseases.

The next feasibility trial for histotripsy translation is in pancreatic cancer, an area of unmet need where the GANNON trial (NCT06282809)^69^ is being conducted in Barcelona to evaluate its safety.^33^ The scientific community and pancreatic cancer patients are eagerly awaiting this trial outcome.

Completed and ongoing trials utilizing the HistoSonics’ Histotripsy Systems are summarized in Table 2.

**Table 2:** Summary of completed and ongoing trials of HistoSonics’ Histotripsy Systems.

Trial	Target organ and population	Study type	Key results/objective
**THERESA (NCT03741088)**	Liver: Patients with unresectable primary and metastatic liver tumours	Phase 1 (completed)	First-in-human trial that demonstrated the safety and feasibility of hepatic histotripsy. Histotripsy can effectively and predictably destroy liver tissue, with treated volumes correlating well with the planned volume. No device-related adverse events were reported.
**#HOPE4LIVER (US & EU) (NCT04572633, NCT04573881)**	Liver: Patients with unresectable primary and metastatic liver tumours	Pivotal (completed)	Histotripsy is safe and effective, meeting primary endpoints. Local control rate of 90% was achieved at 12 months. This provides evidence for FDA Clearance in October 2023 for the non-invasive destruction of liver tumours.
**BOOMBOX: Master Study (NCT06486454)**	Liver: Patients with primary and metastatic liver cancer or benign liver tumours	Post-Market Registry (ongoing)	To collect real-world data on the use of the Edison system for treating liver tumours following its FDA clearance. This provides further insights into the technology’s performance and long-term outcomes in a broader patient population.
**CAIN (NCT05432232)**	Kidney: Patients with non-metastatic solid renal tumour ≤3 cm	Phase 1 (completed)	A first-in-human study to evaluate the safety and feasibility of histotripsy for the non-invasive ablation of kidney tumours. Results awaited.
**#HOPE4KIDNEY (NCT05820087)**	Kidney: Patients with non-metastatic solid renal tumour ≤3 cm	Pivotal (completed enrolment)	Building on the CAIN trial, this study aims to evaluate the safety and efficacy of histotripsy for destroying targeted kidney tumours, to support a future regulatory submission for a kidney indication.
**GANNON (NCT06282809)**	Pancreas: Patients with unresectable locally advanced or oligometastatic pancreatic adenocarcinoma.	Phase 1 (ongoing)	To evaluate the safety of the HistoSonics Edison System for the destruction of pancreatic tumours.

## Potential challenges in early adoption of histotripsy in clinical practice

While early adoption of histotripsy in the United Kingdom has so far been embraced in early phase trials, there remains a variety of technical, clinical, economic, regulatory, and cultural barriers to the full adoption of histotripsy in the NHS practice.

### Technical barriers

Histotripsy relies on continuous, direct visualization of the target lesion under ultrasound, which can be limited by respiratory movement, acoustic shadowing from ribs, artefact from bowel gas and a high body mass index, depending on the location of the target lesion.^52–55^ Current liver and renal histotripsy protocols require general anaesthesia to limit respiratory movement.^53^ In the THERESA study, assessing the feasibility of the treatment for liver lesions (which used a previous version of the Edison™ system), 21% of patients were excluded for inadequate treatment windows, and posterior-superior segment lesions were not attempted.^61^ In pre-clinical studies assessing the feasibility of pancreatic tumour treatment in a porcine model, extensive bowel preparation was required to facilitate an acoustic window, which was not always successful.^6^ This has, however, significantly improved since the introduction of the current latest Edison™ system, where the ultrasound probe can be lowered from the treatment head to improve localisation of the targeted tumour. The Edison™ system also uses image fusion capability to allow less well-visualized lesion to be targeted with fusion software.

Despite the use of general anaesthesia, the ultrasound beam can defocus during treatment, requiring constant operator input, monitoring the accurate contour from treatment planning and the cavitation process in real-time.^61^ A learning curve is also to be expected, as the monitoring of histotripsy is only partially transferable from other ablative treatment methods. The set-up and treatment planning are also very different to conventional ablative techniques, including the placement of the water bath, ultrasound probe, patient positioning, and the technical aspects of the software system. One centre has reported an approximate room occupancy time of 2-4 hours per histotripsy procedure.^70^ Credentialling frameworks and multi-centre registries will be essential to standardise treatment protocols and outcomes.

### Clinical and regulatory barriers

Histotripsy is an innovative medical technology, and early adoption of this technology in the UK has posed a multitude of challenges due to the evidence gap. While randomized control trials such as the landmark COLLISION trial,^71^ EORTC 40004 trial,^72^ and the SURF trial^73^ have shown that thermal ablation is non-inferior to chemotherapy or surgical resection for colorectal liver metastases and primary hepatocellular carcinoma (HCC), respectively, there is currently a lack of robust long-term oncological endpoints such as local-recurrence-free survival and disease-specific survival for histotripsy. This lack of evidence hinders wider NHS adoption and commissioning of histotripsy. This is because UK-specific data are currently limited despite our leading role in international trials. Healthcare systems may vary significantly in structure, patient population, and standard treatment approaches, making it important to demonstrate effectiveness specifically within the NHS context. This is likely to require UK-led studies or registries to first gather real-world evidence that reflects local practice patterns and outcomes. The National Institute for Health and Care Research (NIHR) and NHS England are, through their joint National Research Collaboration programme, exploring what would be needed to prioritise a multi-site comparative study, aiming to generate the evidence required for the routine commissioning of histotripsy in the NHS. This will help ensure more diversified demographics data and accelerate the evidence journey, connecting multiple NHS sites in a joint-up effort with stronger synthesis. In September 2025, NIHR Health Technology Assessment commissioned a funding call for the Histotripsy Edison System, and it would be exciting in the coming years, pending on the outcome of this funding opportunity, especially the impact for patients in the early evaluation of this technology in the UK NHS landscape.

This gap in clinical evidence is reflected in the current regulatory status of the Edison™ system in the United Kingdom, with only limited market access approved by the MHRA, and a lack of NICE Medical Technology Guidance for histotripsy. Clinicians must operate within a clinical trial framework until NICE or the NHS issues further guidance. To bridge this evidence gap, UK researchers must promptly publish mature trial results and organize multi-centre studies, demonstrating not only technical success and safety, but also oncological, quality of life, and economic viability for histotripsy.

At present, histotripsy may be limited to tumours that are not eligible for conventional ablation strategies, or to those patients specifically requesting a non-invasive treatment option. In the case of the latter, informed consent must emphasize the current evidence for this treatment and the unknown long-term efficacy.

### Economic barriers

As an expensive new technology, costing around £1.5 million per treatment machine, histotripsy requires a large initial capital expenditure and ongoing costs. In addition to the established clinical role and evidence base, the endorsement by national organizations, for example, NICE, will be highly dependent on the cost-effectiveness compared to the existing standard of care. Only after national endorsement, histotripsy would be locally commissioned for wider adoption in individual NHS centres.

It can be postulated that the HistoSonics Edison™ system may be cost-effective due to its non-invasive nature, possibly allowing it to be a day-case cancer treatment, avoiding long hospital stays and sequelae of complications. While not directly compared to other modalities, the postulated lower complication rates^10^ and quicker recovery times might also shorten follow-up costs. Furthermore, the Edison™ system’s mobility allows for treatment without a dedicated treatment room, offering flexibility in location. This flexibility could potentially reduce opportunity costs by freeing treatment rooms to enable other critical interventions to be performed.

The NHS commissioning framework would require a cost-effectiveness analysis to show that the Edison™ system provides at least an equivalent outcome at a lower or acceptable overall cost. To date, no NICE guidance or tariff codes exist for histotripsy for NHS service commissioning. There needs to be an innovative pathway approach for early Medtech adoption in the NHS, as philanthropy and research grants alone will not be a sustainable approach for full early adoption and provision in the long term. The maintenance and upkeep costs of the unit are non-negligible and currently difficult to estimate. Ultimately, demonstrable health economic benefits (eg, cost per quality-adjusted life-year) will be needed to convince NHS funders. This requires not only accounting for device cost but also modelling long-term outcomes, complication avoidance, and comparisons with costs for standard locoregional therapies or providing a break from systemic therapy.

### Clinician and human factors

Interventional Radiology thrives on innovation, but convincing experienced interventionalists to adopt new tools can be challenging, especially when teams have established conventional ablation practices and workflows. Existing ablation methods already offer swift recovery with minimal morbidity, prompting questions about the necessity of new approaches. This is especially true given the lack of evidence of long-term benefit. For the leader who is leading the positive disruptive change, it is vital to empower and engage the frontline clinicians and teams to understand the reasons why we are adopting the innovation. For example, there are benefits for patients who might favour non-invasive (needle-less) options if they offer even better tolerability. Understanding the bigger picture helps others to embrace change and be willing to consider the adoption of a new approach. Furthermore, while conventional ablation may seem suitable for easy-to-treat lesions, using histotripsy for simpler cases first can demonstrate its feasibility and pave the way for broader adoption. Ultimately, long-term evidence of oncological outcomes and cost-effectiveness is crucial to drive multi-disciplinary team (MDT) adoption of new methods. Additionally, histotripsy offers the potential advantage of ablating less accessible tumours or tumours in close proximity to other structures and vasculatures.^25^ With increased clinical trial evidence, confidence in the technology within the MDT will enable adoption into standard clinical practice.

### Patient factors

Patients often struggle to keep up with new healthcare technologies due to complex terminologies, the rapidly evolving landscape, and difficulty in finding reliable information. It is likely that public awareness of histotripsy, as a novel technique, is limited. This knowledge gap may prevent patients from fully participating in shared decision-making regarding their treatment options, potentially posing a barrier to clinical trial participation. However, the positive outcomes of the #HOPE4LIVER trial and the world’s first kidney case in the CAIN trial have significantly improved public awareness, generating substantial publicity in the form of news, videos, and blogs from the public.^62^^,^^74–76^ Furthermore, data gathered from the NIHR Participation in Research Experience Survey evidenced entirely positive patient feedback for all participants in the CAIN trial.^77^ For all patients, this was the first clinical trial they had participated in, and they felt well prepared for the trial by the information received in the consent process, and all stated that they would take part in clinical research in the future.

Following these results and the easy availability of information online, UK institutions involved now receive numerous inquiries from patients seeking to undergo this revolutionary treatment. However, patients currently must travel to the United Arab Emirates or the United States at their own expense to receive this treatment outside of any existing trial context. Furthermore, during the early adoption phase, histotripsy may only be available at selected UK centres, potentially creating geographic disparities in access. To mitigate this, clear criteria for patient selection are needed to ensure that only those most likely to benefit receive treatment.

The novelty of histotripsy complicates informed consent. Clinicians must effectively communicate the procedure’s known benefits, risks, and uncertainties, especially regarding long-term outcomes, while acknowledging the evolving evidence base. This means providing balanced information to set realistic expectations, avoiding both undue pessimism and excessive optimism.

## Opportunities and future directions

Despite the challenges outlined, histotripsy presents numerous opportunities for advancing patient care and clinical practice in the United Kingdom. Several key areas of opportunity merit exploration and development as the technology continues its trajectory toward broader clinical implementation.

### Expansion of clinical applications

While liver tumours represent the first approved application for histotripsy, the technology’s potential extends to numerous other scenarios. Pre-clinically, notable investigations in animal models are ongoing for intracranial tumours,^78^ sarcomas,^79^ cholangiocarcinomas^80^ and prostate cancer.^59^ Human trials of these tumours are eagerly awaited.

The role of histotripsy in combination with immune-checkpoint inhibitors in the advanced and metastatic disease setting through its immunomodulatory properties has also been explored through pre-clinical experiments. The mechanical and non-thermal disruption of cancer cells from histotripsy preserves tumour-associated antigens, which are reabsorbed by the body over several weeks post-histotripsy. These antigens, along with damage-associated molecular patterns and inflammatory cytokines, activate the adaptive immune system, allowing for priming of antigen-specific T cells. The tumour-antigen-specific T cells can then travel in systemic circulation to reach untreated tumours, allowing direct destruction of local and distant tumour cells, resulting in an abscopal effect.^34^^,^^81^ This was demonstrated partially in a patient with colorectal liver metastases from the THERESA feasibility study of histotripsy in liver tumours.^61^^,^^82^ Sustained response leading to complete regression of tumours has yet to be observed and requires further investigation. There have been promising results from a murine model showing complete regression of contralateral neuroblastomas after treatment of the primary tumour.^33^ This anecdotal evidence suggests that the combination of immunotherapies with histotripsy holds the potential to greatly amplify immune anti-cancer responses, inducing an adjuvant effect to treat micrometastatic disease in potentially curative cases, and improving efficacy in non-curative cases.

In benign diseases, pre-clinical and early clinical studies show potential applications of histotripsy in cases such as deep vein thrombosis and pulmonary embolism, urinary-tract calculi,^83^^,^^84^ surgical site infections,^85^ intracerebral haemorrhage,^86^ mitral stenosis,^87^ aortic stenosis,^88^ and uterine fibroids.^89^ Each of these potential applications represents an opportunity to address unmet clinical needs within the NHS and improve outcomes for patient populations with limited treatment options. UK institutions, with their strong research infrastructure and multi-disciplinary expertise, are well-positioned to lead some of these expansion efforts.

### Integration with NHS cancer pathways

The NHS Long Term Plan^9^ emphasizes earlier cancer diagnosis and more effective treatment options as key priorities. Histotripsy could potentially support these goals by providing a less invasive and more cost-effective option for patients diagnosed with cancer. In the United Kingdom, liver cancer currently ranks as the eighth most common cause of cancer-related deaths, accounting for approximately 3% of all cancer fatalities.^90^ For histotripsy to be integrated effectively into these cancer pathways, several developments are warranted.

Firstly, histotripsy must be incorporated into relevant NICE guidelines and care pathways. Partnership of the public and private sectors will be pivotal in negotiating decisions regarding funding, commissioning, and the development of national guidelines.

After the incorporation of histotripsy into relevant guidelines, referral mechanisms and pathways must be developed to ensure efficient and effective multi-disciplinary discussion of treatment. Establishing MDT protocols and criteria for patient selection and follow-up, as well as standardised treatment protocols specific to the NHS context, is prudent.

Early engagement with NHS England and local trusts will be vital to demonstrate the potential benefits and cost-effectiveness of histotripsy. Secondly, it is key to collaborate with industry partners to ensure device availability, ongoing technical support and training along with fostering new research. Histotripsy easily fits into existing cancer pathways for tumour ablation, meaning the technology can be rapidly established in standard care of practice.

### Building centres of excellence and international collaboration

The early experience with histotripsy at LTHT and NuTH provides a foundation for establishing UK centres of excellence for histotripsy, where treatment protocols can be co-developed with technical guidance for dissemination to other centres. Further research and training in histotripsy can also be carried out in these centres.

The UK’s early involvement in histotripsy clinical trials positions it well for continued international collaboration in this field. Partnerships with centres in the United States, Europe, Hong Kong^91^ and Singapore^92^ could accelerate knowledge exchange, facilitate multi-centre studies, and enable shared learning as the technology matures. Allowing collaboration internationally to share knowledge and best practices will be invaluable to accelerate the learning curve for histotripsy implementation and help establish the UK as a global leader in this emerging field.

### Workforce development and training

While not necessarily a limitation, implementing a new technology such as histotripsy requires establishing an efficient procedural workflow and training MDT, including but not limited to radiologists, surgeons, oncologists, anaesthetists, sonographers, and nurses. Dedicated training programs and proctoring will be necessary to ensure safe and effective implementation. The time it takes to build the experience in treatment planning, device operation and post-procedure management can therefore arguably be seen as a limitation. However, in the long term, this will aid the establishment of competency frameworks and credentialling processes, which are essential for safety and quality control. The collaborative working with centres of excellence will help to incorporate histotripsy into training curricula of the Royal College of Radiologists, European Board of Interventional Radiology, and the Institute of Physics and Engineering in Medicine. These workforce development initiatives would ensure that, as histotripsy becomes more widely available, appropriately trained professionals are ready to implement it safely and effectively.

### Research and registry development

The UK’s robust clinical research infrastructure serves as a solid foundation for addressing the knowledge gaps of long-term oncological outcomes with histotripsy, the comparative effectiveness of histotripsy in relation to conventional treatments, and the conduct of health economic analyses tailored to the NHS context. It is also important that routine quality assurance is performed under Medical Physics and Clinical Engineering guidelines to ensure the safety of histotripsy devices.

In the context of advanced or metastatic disease settings, future research could concentrate on the synergistic effects of histotripsy and immunotherapy, particularly in high-risk patient groups. In the case of advanced or metastatic HCC, the combination of histotripsy and atezolizumab plus bevacizumab may prove beneficial. Similarly, in advanced or metastatic renal cell carcinoma, the potential application of histotripsy to enhance immunogenicity in conjunction with nivolumab and ipilimumab is noteworthy.

In the context of local disease, comparative studies with current curative treatment options are of paramount importance. A direct head-to-head trial is indispensable in ascertaining the efficacy and cost-effectiveness of histotripsy. Moreover, the integration of advanced imaging modalities and artificial intelligence is poised to further transform histotripsy. Machine learning algorithms could perhaps facilitate real-time monitoring of the cavitation process, adapt to changes in tissue properties and optimize the resulting ablation zone, potentially improving safety margins.^17^

Furthermore, it is important to establish a comprehensive national and international registry of cases involving histotripsy, which will serve as a valuable resource for reflective analysis and evaluation of histotripsy in the future. Building on the IDAP Pilot program, securing funding and investment for clinical research trials and devices (beyond industry-sponsored studies will be critical. This may involve seeking grants from national research bodies, eg, NIHR), cancer charities (eg, Cancer Research UK), and exploring opportunities for public-private partnerships to share the financial burden of adoption. The IDAP Program represents a positive step in facilitating access and potentially supporting funding pathways for innovative technologies such as histotripsy.

## Conclusion

The UK’s path to widespread adoption of image-guided histotripsy presents both transformative opportunities and significant challenges. Initial trial results are promising, positioning it as a powerful tool for unmet clinical needs. However, hurdles remain, including the need for robust long-term oncological data, comparative research against existing treatments, the high initial cost of the system, and complexities in workforce training and clinical integration. Ensuring equitable patient access and clear communication about this novel therapy are also crucial. A multi-disciplinary approach and continued collaboration between Trusts, academic institutions, industry and government will be essential to realize histotripsy’s full potential in the UK healthcare landscape. Supporting health economic analyses, continuous rigorous research and establishment of dedicated centres of excellence will allow effective adoption of histotripsy to advance cancer care.
